# Development and application of rTMS device to murine model

**DOI:** 10.1038/s41598-023-32646-w

**Published:** 2023-04-04

**Authors:** Jin Seung Choung, Sohom Bhattacharjee, Jeong Pyo Son, Jong Moon Kim, Dong Sik Cho, Choon Sik Cho, MinYoung Kim

**Affiliations:** 1 Department of Rehabilitation Medicine, CHA Bundang Medical Center, CHA University School of Medicine, 59 Yatap-ro, Bundang-gu, Seongnam, Gyeonggi-do 13496 Republic of Korea; 2 Department of Biomedical Science, CHA University, Seongnam, Republic of Korea; 3 Rehabilitation and Regeneration Research Center, CHA University School of Medicine, Seongnam, Republic of Korea; 4 School of Electronic and Information Engineering, Korea Aerospace University, 76, Hanggongdaehak-ro, Goyang-si, Gyeonggi-do 10540 Republic of Korea; 5 R&D Center, Remed Co., Ltd., Seongnam, Republic of Korea; 6 Advanced Radiation Technology Institute (ARTI), Korea Atomic Energy Research Institute (KAERI), Jeongeup, Republic of Korea

**Keywords:** Biophysical models, Biomedical engineering

## Abstract

Repetitive transcranial magnetic stimulation (rTMS) is attracting attention as a new treatment technique for brain lesions, and many animal studies showing its effects have been reported. However, the findings of animal application researches cannot directly represent the effects of rTMS in human, mainly due to size difference and mechanistic characteristics of rTMS. Therefore, the authors purposed to develop a mouse rTMS to simulate clinical application and to confirm. Firstly, a virtual head model was created according to magnetic resonance images of murine head. Then, simulations of rTMS stimulation with different coils were performed on the murine head phantom, and an rTMS device for mice was fabricated based on the optimal voltage conditions. Lastly, strengths of magnetic fields generated by the two rTMS devices, for human (conventional clinical use) and mouse (newly fabricated), were measured in air and on mouse head and compared. Resultantly, the magnetic field intensity generated by coil of mouse was lower than human’s (*p* < 0.01), and no differences were found between the predicted simulation values and the measured intensity in vivo (*p* > 0.05). Further in vivo researches using miniaturized rTMS devices for murine head should be followed to be more meaningful for human.

## Introduction

Transcranial magnetic stimulation (TMS) is a technique that noninvasively modulates brain activity using magnetically induced electric fields^1^. It has received US Food and Drug Administration approval for cortical mapping, has been used to treat several psychiatric and neurological disorders, and is being studied for use in many other conditions^2^. Significant therapeutic efficacy was recently reported for degenerative brain diseases, such as stroke and dementia, and therapeutic efficacy has been revealed as a mechanism for stimulating brain nerve cells and increase neuroplasticity^3,4^.

Although the cellular and molecular mechanisms underlying repetitive TMS (rTMS)–induced neuronal recovery have been systematically studied in rodent models, suitable rTMS coils for rodents are lacking^5^. Thus, rTMS coils for rodents have been developed and used, but most used commercially available human coils^6^; therefore, our understanding is limited of research on accurate nerve stimulation. Developing a small rTMS coil suitable for rodents is difficult because of increased resistance, overheating, and coil rupture, but brain stimulation can theoretically be focused more precisely^7^. Other studies have shown that rodent-specific rTMS coils with reduced stimulation intensity are more focal^8,9^; however, the results induced by low-intensity stimulation coils are not representative of those induced by high-intensity stimulation coils used in human rTMS studies^10^.

The spatial and temporal parameters activated by rTMS, as well as coil design, have not been studied considering the complexity of geometries within the brain, and insightful studies of the origin and mechanism of physiological responses are lacking^11^. In particular, although there have been many modeling studies on humans, modeling studies of experimental animals such as mice and rats are insufficient, which causes differences between clinical and basic scientific research. The therapeutic efficacy of rTMS has been reported in many animal models, whose results were much more dramatic than those reported by clinical studies. However, reports are lacking on the differences between clinical and animal head models^12^.

Computational modeling is a powerful tool for investigating the mechanisms of TMS and identifying the stimulus parameters. Previous modeling studies focused on calculating the spatial distribution of the electromagnetic field induced by TMS using the finite element analysis method (FEM) in a head model derived from magnetic resonance imaging (MRI) data^13,14^. However, the development of novel TMS treatment procedures for neurological and psychiatric disorders using human subjects or animal models has several ethical and technical limitations. As TMS trials using human patients are not always possible, brain phantoms have been developed to test experimental setups without stimulating patients. The use of phantoms further allows for experimental validation of TMS stimulation for both induced electric fields and voltages^15^. Brain phantoms for rodents designed specifically for neuromodulation techniques have been reported. Individualized brain phantoms for rodents are required to accelerate the study of neuromodulation techniques, particularly the measurement of induced electric fields and voltages in brain regions during rTMS^16^. Models for rodent brain regions have been proposed; however, it is unknown whether they include simulations for elements such as the skull and cerebrospinal fluid^17^.

In this study, rTMS coils optimized for animals were created by conducting simulations considering various TMS coil shapes, angles, and strengths. Moreover, to determine the magnetic field strength in the brain using rTMS, a geometrical model of the cortex of the experimental animal was constructed based on MRI data. The magnetic field applied by the rTMS developed using a Tesla meter was measured on the head of the experimental animal and compared with the simulation results.

## Methods

### Coil design theory

**Figure 1:**
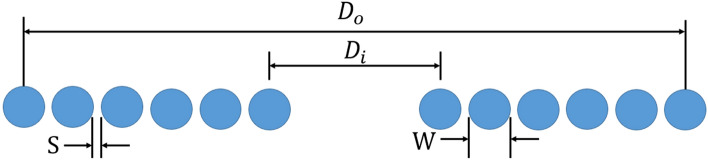
Physical dimensions of the Harold–Wheeler formula.

Before designing the coil, it is important to calculate the required parameters such as coil inductance and H field intensity so we can use the minimum turns and optimal design to reach the required values. The inductance and magnetic field intensity were calculated mathematically. The Harold–Wheeler formula was used to calculate the inductance^13,14^. This formula is applied at "low" frequencies (< 30 MHz) using enameled copper wire. The inductance (L) can be calculated as follows:

1 $$L = \frac{{N^{2} A^{2} }}{{30A - 11D_{i} }}$$

where *D*_*i*_ denotes the inner diameter of the coil set to 20 mm and N and A represent the number of turns and cross-sectional area of each turn of the coil, respectively (Fig. 1). Further, the area of the coil can be calculated as

2 $$A = \frac{{D_{i} + N(W + S)}}{2}$$

where *W* denotes the width of the wire (considered 3 to maintain a smaller size), and *S* denotes the spacing between the coil's turns, which is set as 0.2 mm. As we required a smaller coil, the turn of the coil (*N*) was kept at 7 to enable the focus most of the H field on the murine brains. Solving Eqs. (1) and (2), the calculated inductance of the coil was 2.04 *μ*H. Furthermore, the H field intensity (B) was calculated as

3 $$B = \frac{{\mu_{0} NI}}{2R}$$

where *I* is the input current of the coil, which is set to 1000 A, and *R* is the total radius of the coil. Therefore, the calculated H field intensity was 0.43 T.

### Simulation

**Figure 2:**
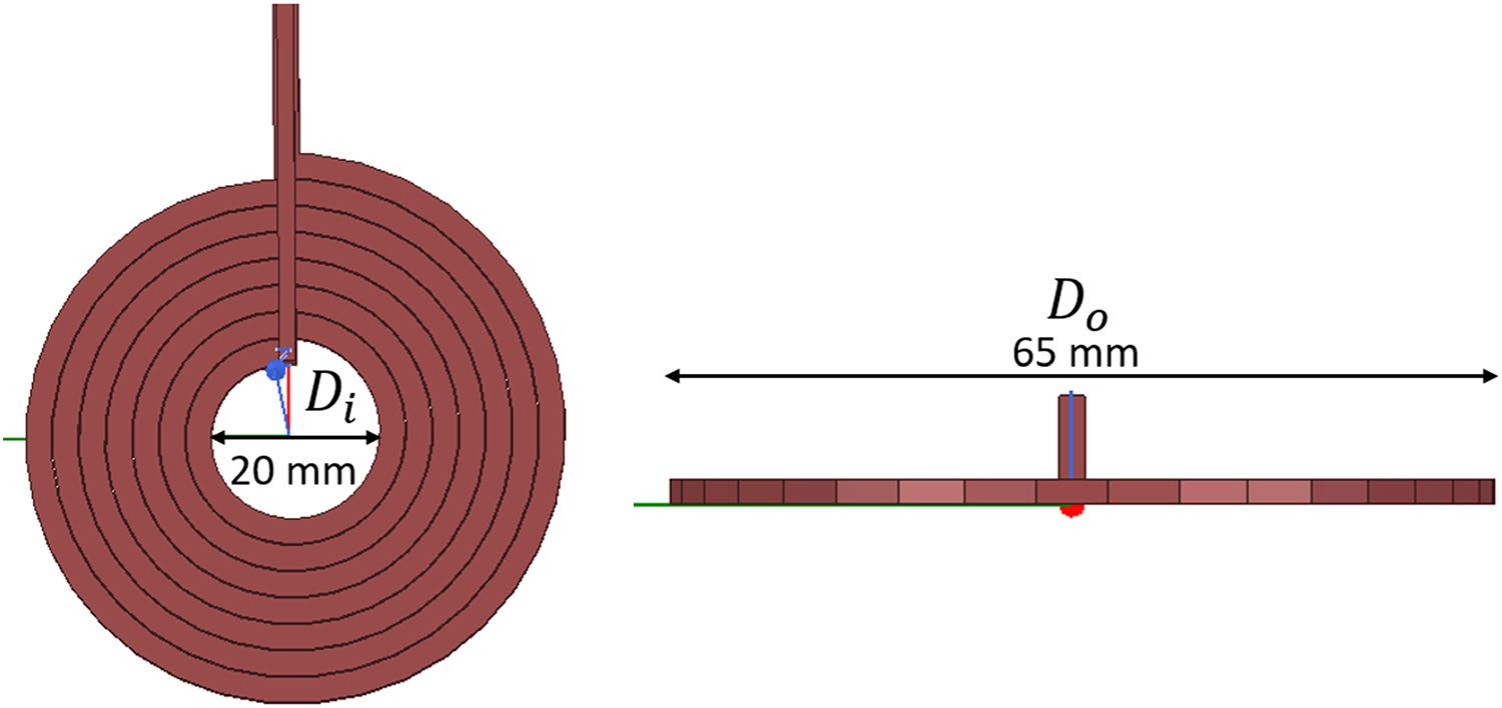
Coil design and parameters.

After calculating its parameters, we designed and analyzed the coil using an FEM simulation tool (Ansys Maxwell®). The frequency of stimulation was conducted with 20 Hz due to its beneficial biological effects on the Alzheimer’s disease model brain^3^. And, for thermal profile analysis, Femtet® (Murata Software Co., Ltd., Tokyo, Japan) was used. The designed coil is shown in Fig. 2.

The design parameters are summarized in Table 1.

**Table 1:** Designed coil parameters.

Width	Height	Turns	Gap between turns	Current	Voltage	Frequency	Inner diameter (*D*_*i*_)	Outer diameter (*D*_*o*_)
3 mm	2 mm	7	0.2 mm	1000 A	500 V	20 Hz	20 mm	65 mm

#### Simulation results

The designed coil was simulated in Ansys Maxwell^®^, and the inductance of the coil was analyzed (Table 2). The calculated result is almost identical to the previously calculated inductance.

**Table 2:** Inductance of the designed coil.

Frequency (Hz)	Inductance (μH) Setup 1: last adaptive
20.0	1.956066

**Figure 3:**
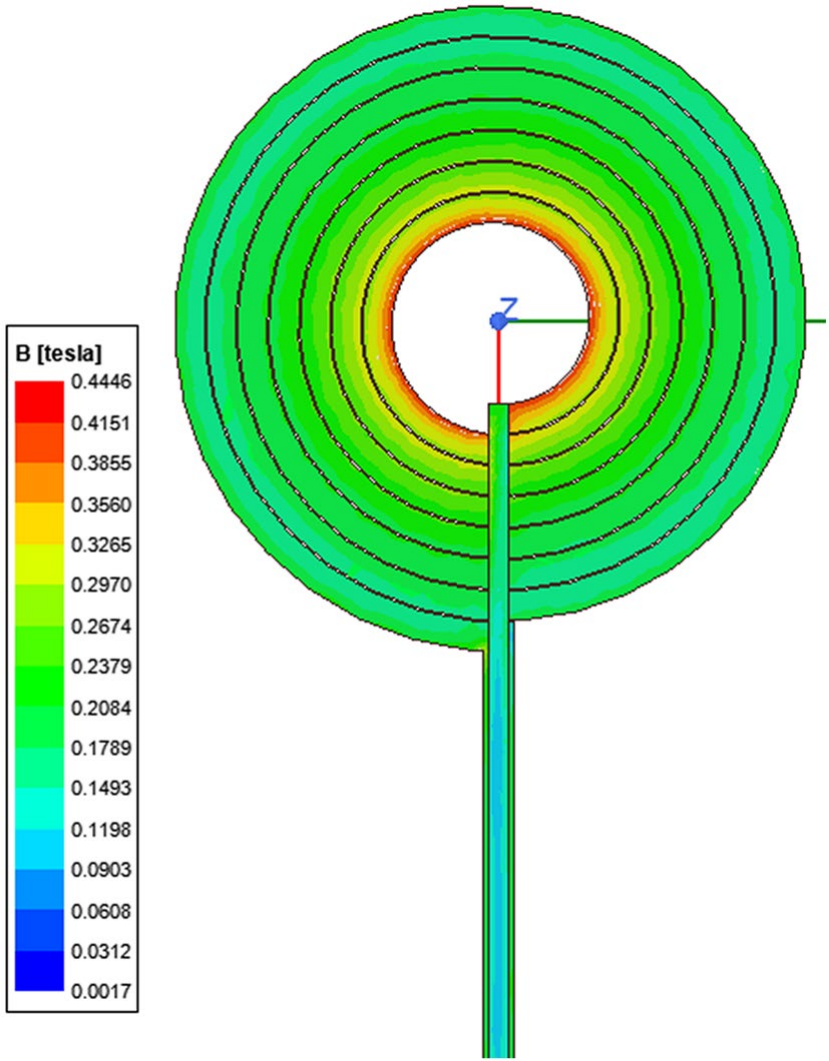
Magnetic field intensity of the designed coil.

Furthermore, the magnetic field intensity on the coil's surface was analyzed and matched with the calculated results shown in Fig. 3. The magnetic field intensity was simulated using Finite element simulation tool with finite element method^11^. Therefore, the magnetic field in this study is the vector sum of the magnetic field intensity. The coil's maximum magnetic field intensity is generated at its center (0.44 T), which matched our calculated value.

#### Coil design comparison

**Figure 4:**
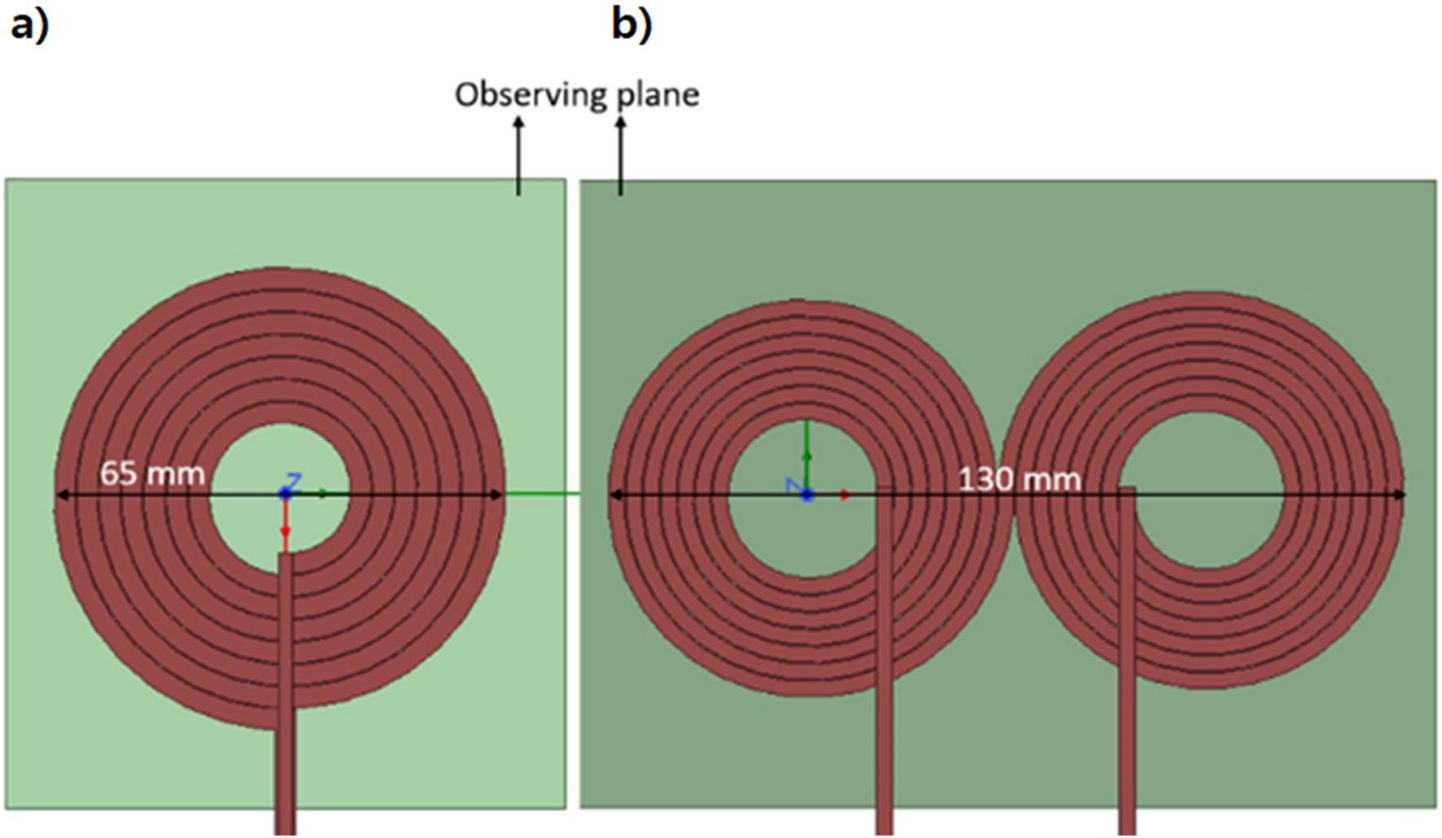
Simulation setup of coils for simulation of magnetic field intensity: (**a**) designed circular coil; (**b**) figure 8-shaped coil.

To design a new rTMS coil, we optimized its design by simulating the thermal stability and focusing degree. The circular coil was adopted in this study because it demonstrates superior fine focusing ability and generates less heat compared to figure 8-shaped coil. To confirm this, we performed several simulations. In the initial stage of this study, we designed a figure 8-shaped coil with similar parameters and kept a reference plane 5 mm apart to compare the magnetic field pattern with circular coil shown in Fig. 4.

**Figure 5:**
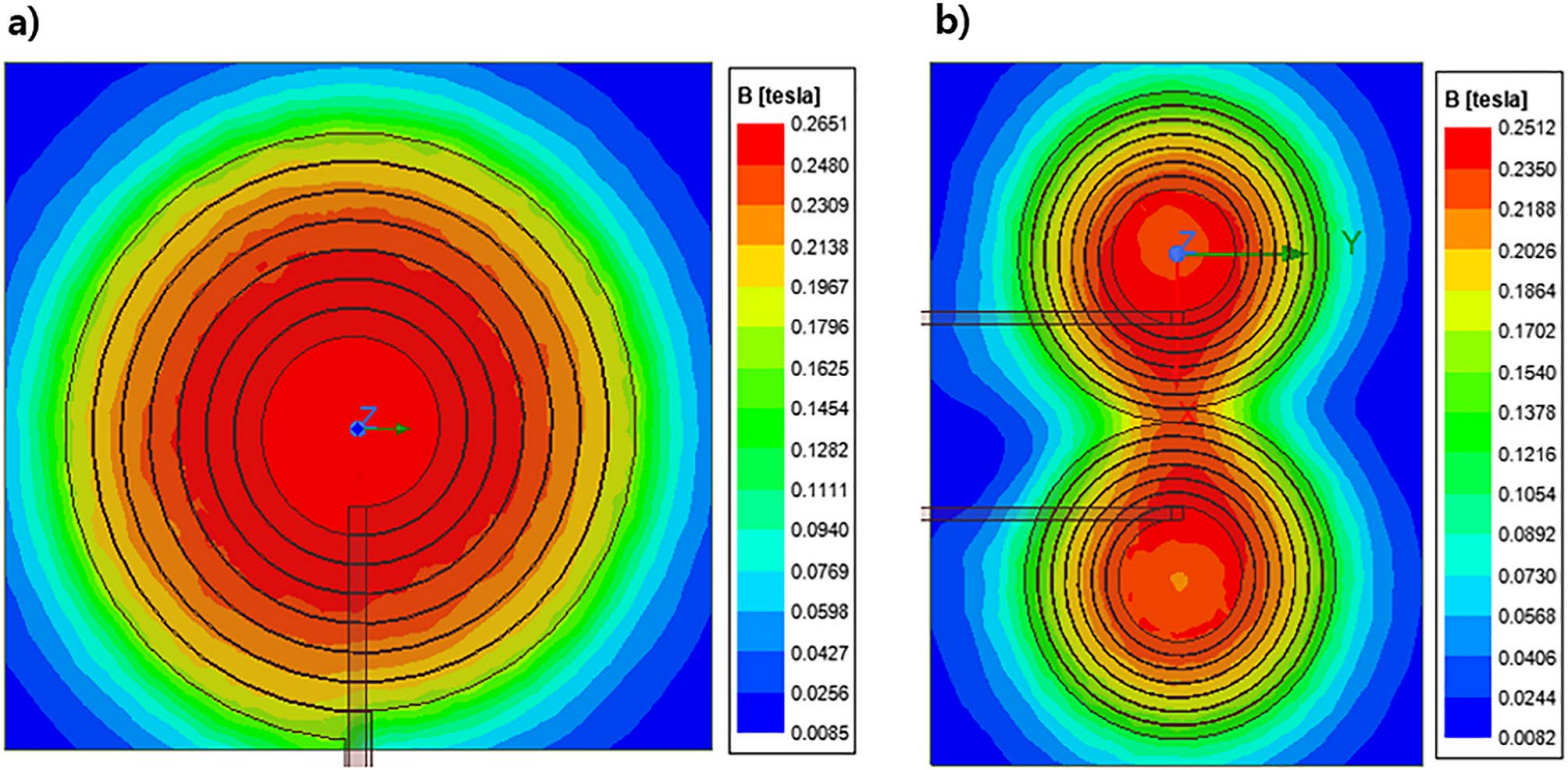
Comparison of magnetic field intensity on observation plane: (**a**) designed circular coil; (**b**) figure 8-shaped coil.

After running the magnetic field analysis simulations, we have observed that figure 8-shaped coil generates two focusing magnetic field patterns when placed close to the subject as shown in Fig. 5b, whereas the circular coil is still producing a single focusing magnetic field pattern when placed close to the subject as shown in Fig. 5a. Also, the magnetic field pattern of figure 8-shaped coil is much wider than circular coil as we are trying to focus on a smaller subject and the extra field generated by figure 8-shaped coil will be not of any use.

**Figure 6:**
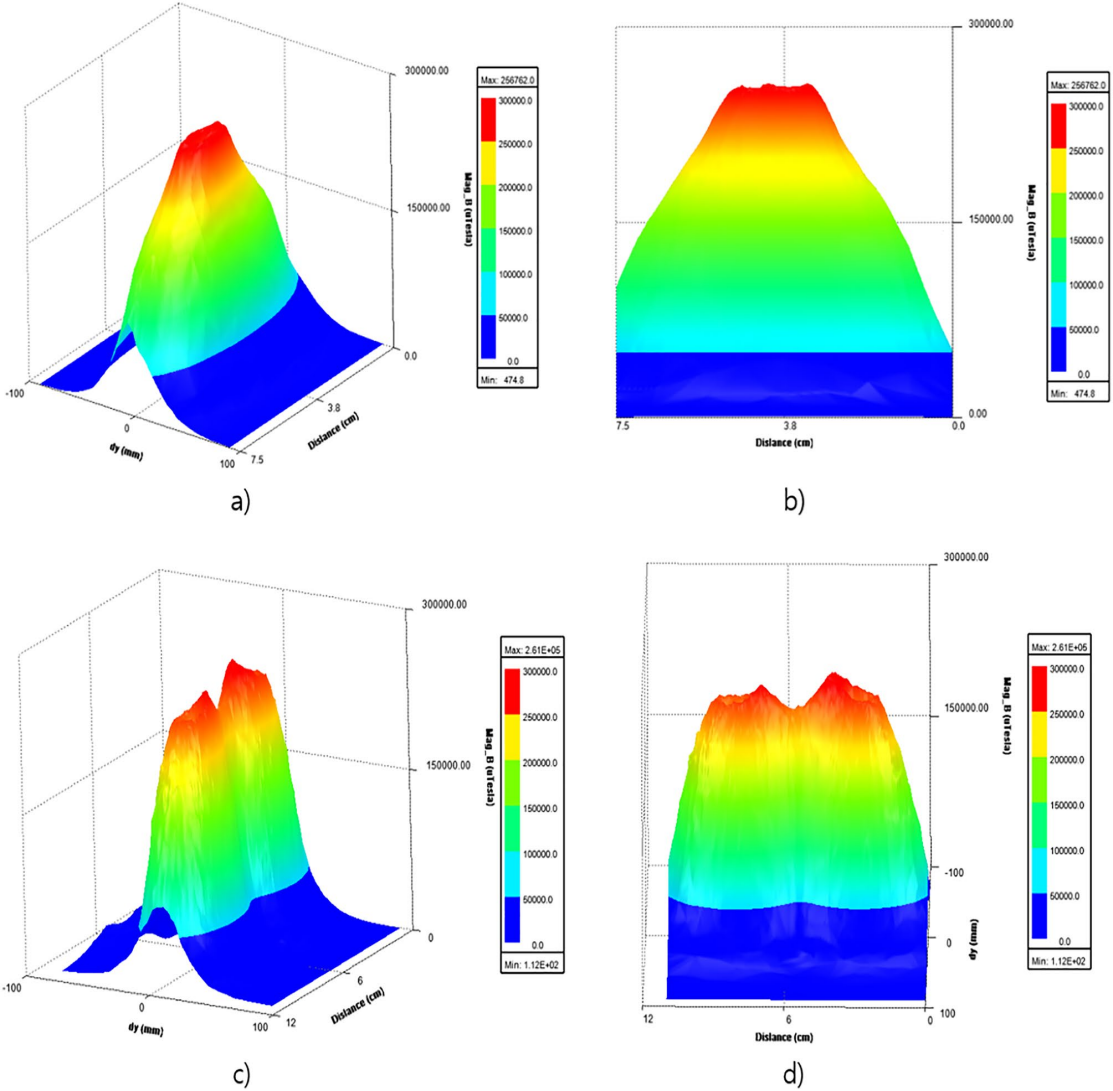
3D plot of magnetic field intensity of (**a** and **b**) Circular coil, (**c** and **d**) Figure 8-shaped coil with respect to distance.

To examine the focus area and quantitative values of focality of two different coils, we performed 3D plot analysis of magnetic field intensity on an observing plane kept 5 mm apart from coil as shown in Fig. 6.

Figure 6a, b shows the 3D magnetic field distribution of circular coil across the observing plane. It is observed that the high intensity field is focused (which is displayed with red in the plot). Whereas Fig. 6c, d shows 3D magnetic field distribution of figure-8 shaped coil across the observing plane where the intensity is not concentrated on a single area, rather it has two different peaks of intensity.

**Figure 7:**
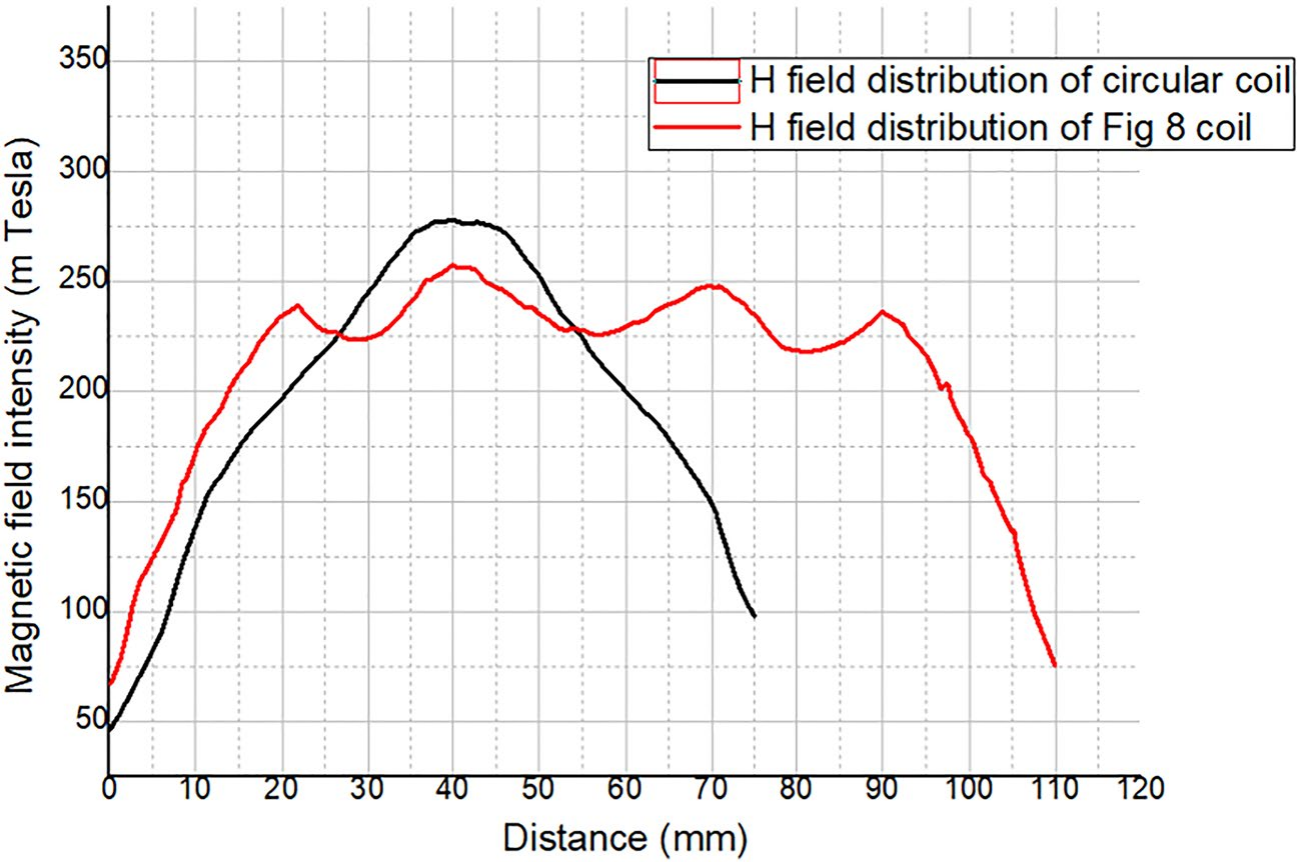
Graphical comparison of magnetic field intensity and pattern of circular coil and figure-8 shaped coil.

**Figure 8:**
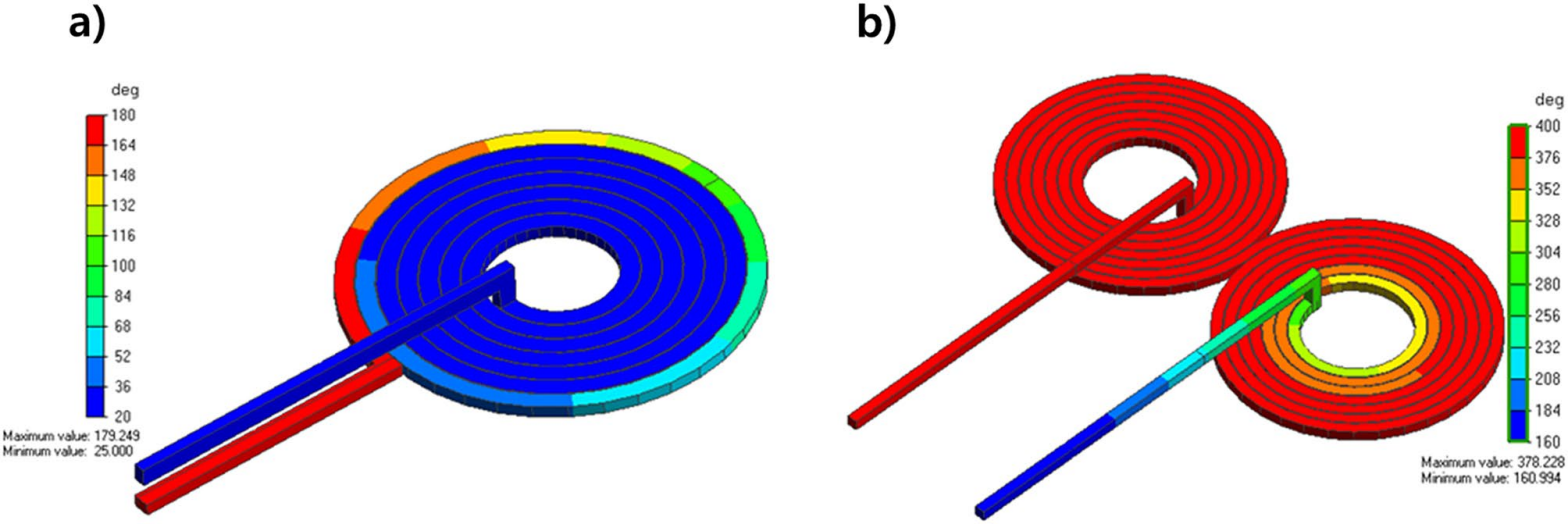
Comparison of thermal analysis of the designed: (**a**) circular coil; and (**b**) figure 8-shaped coil.

Further, the magnetic field distribution of two different coils is plotted on a graph for better comparison of focality as shown in Fig. 7. Therefore, it is concluded that circular coil has more concentrated focusing ability than figure 8-shaped coil.

The circular coil had a better focus than the figure 8-shaped coil. Moreover, the intensity of the circular coil was higher than that of the figure 8-shaped coil. The major drawback of the figure 8-shaped coil is that it focuses on two different places that reduce the magnetic field intensity. Therefore, a circular coil was selected for the experiment. Moreover, it is important to maintain the thermal stability of the rTMS coil. So we further studied the thermal profile of two different coils as our aim was to avoid excess heating produced by the coil. After running the temperature analysis simulations, we found out that figure 8-shaped coil is generating more heat than circular coil because of higher amount of turns as shown in Fig. 6. Thermal analysis of the coil was simulated using Femtet^®^ (Fig. 8). A remarkable difference in temperature was noted between the two coils. Accordingly, we concluded that circular coil is more efficient in terms of magnetic field focusing and thermal stability when the subject is small and close to the coil.

### Murine head structure analysis and simulation model construction

**Figure 9:**
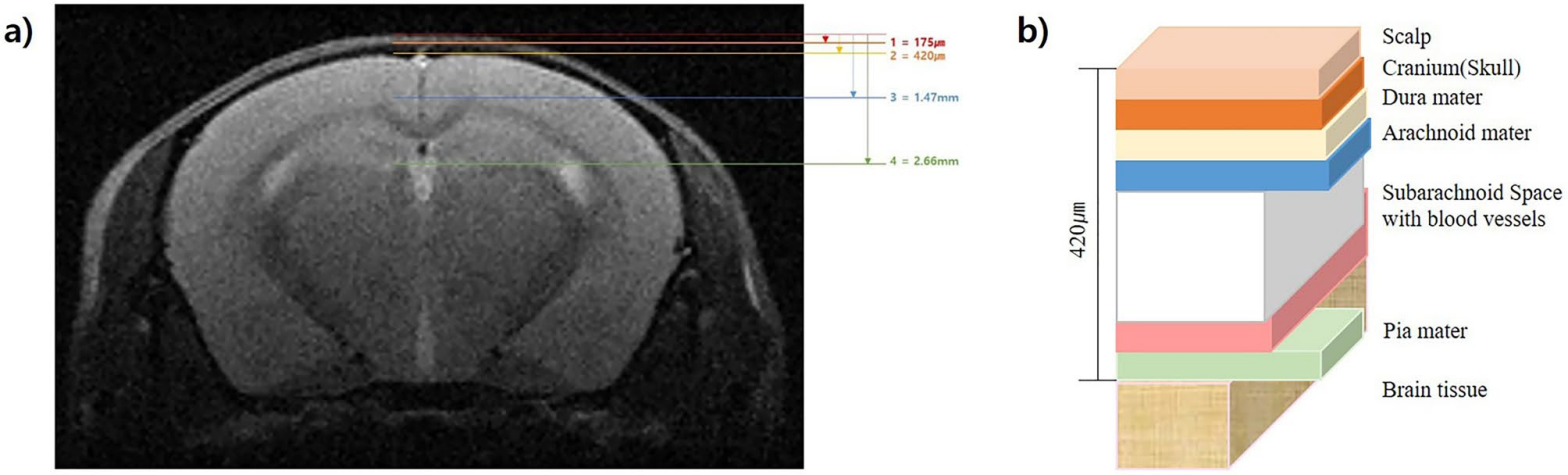
Murine head structure analysis and simulation model construction: (**a**) brain MRI images of C57BL/6 mice; (**b**) analytical model of different layers (scalp, skull, dura matter, arachnoid matter, subarachnoid space, pia matter, brain) of the murine head.

Before using the fabricated rTMS coil, it was necessary to calculate the optimal magnetic field discharge conditions. A head model was required to interpret the simulation results. There are many reports on human models but few on rodents, especially murine head models. Therefore, brain imaging images of C57BL6 mice were obtained using 7.0 T MRI (Fig. 9a). After the depths of the various layers constituting the head were measured, a real murine brain model was simulated (Fig. 9b).

#### Circular coil simulations

**Figure 10:**
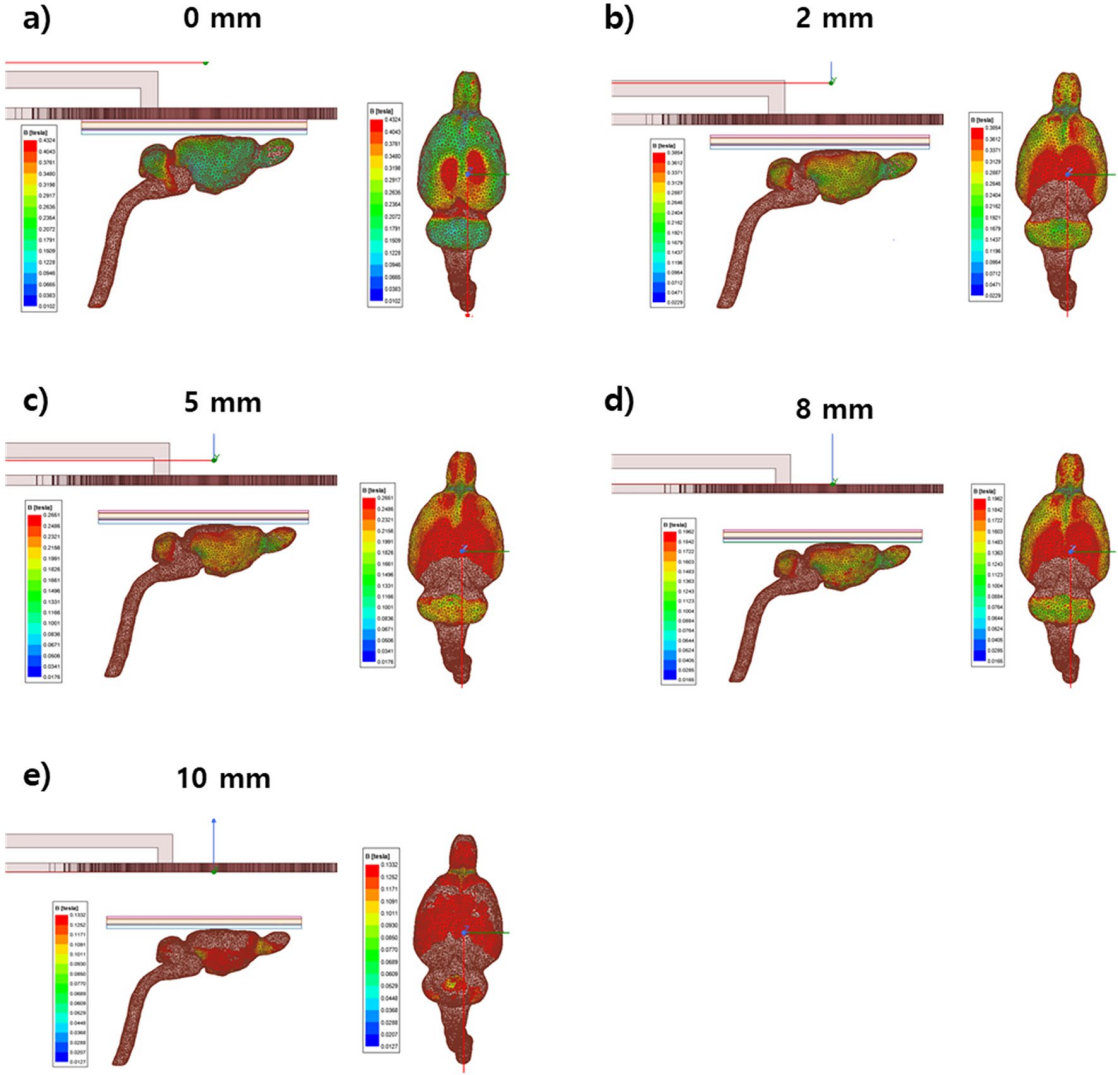
Magnetic field intensity simulation using the mice brain model. The distance between the coil and brain model varied: (**a**) 0 mm apart; (**b**) 2 mm apart; (**c**) 5 mm apart; (**d**) 8 mm apart; and (**e**) 10 mm apart.

Furthermore, a circular coil was simulated using a murine brain model. The objective was to study the change in magnetic field intensity of the coil induced to the murine brain with change in distance, so the coil position was varied (0, 2, 5, 8, and 10 mm away from the model) and the readings were recorded. The magnetic field simulation results are shown in Fig. 10 and Table 3.

**Table 3:** Magnetic field intensity summary.

Distance between coil and murine head (mm)	H field intensity (T)
0	0.4836
2	0.3854
5	0.2651
8	0.1962
10	0.1332

With increasing distance, the magnetic field induced in the brain decreased. The maximum H field was obtained when there was no gap between the coil and the mouse head. The best reading was obtained 2 mm apart, and the intensity of the H field was sufficient to stimulate the brain cells.

**Figure 11:**
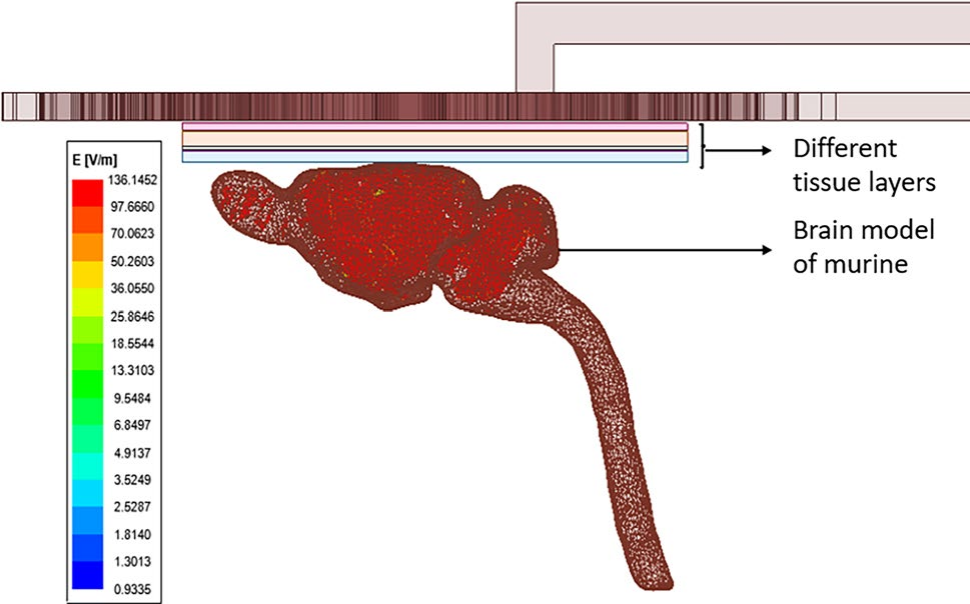
Simulation result of electric field intensity of murine brain model.

Since the conductivity of different tissues is different, according to the MRI data, electromagnetic properties and thickness were fed in different tissues of murine brain model in the simulation tool. The thickness and electromagnetic properties of different tissue layers are summarized in the Table 4. The murine head model was modeled using 6 different tissue layers (scalp, skull, dura matter, arachnoid matter, and brain). According to measurement of the electric field strength on murine brain model, it was obtained as 136.14 V/m (Fig. 11).

**Table 4:** Thickness and electromagnetic properties of the tissues of Murine brain.

Tissue	Thickness (μm)	Permittivity	Conductivity (S/m)
Scalp	500	3056	0.0009
Skull	1000	1246	0.0203
Dura matter	300	2360	0.5010
Arachnoid	75	3013	0.0650
Brain	890	6683	0.1056

### Animal experiments and measurement of magnetic field intensities

All animal experiments were approved by the Institutional Animal Care and Use Committee of CHA University (IACUC210116). Isoflurane was administered via a VEVO COMPACT ANESTHESIA SYSTEM, and anesthesia induction was performed by positioning the nose of each mouse into a small nose cone delivering 3% isoflurane in pure medical oxygen. Anesthetized animals were fixed stereotaxically and rTMS stimulation was applied. After shaving the mouse's neck and making a minimal incision, a Tesla meter (FW Bell's model 8010) probe was inserted into the skull to measure the Tesla under the skull^18^. The mice were housed in four cages and maintained on a daily 12:12 h light–dark cycle in a temperature-controlled room. The animals were provided standard rodent food and water ad libitum. The mice were allowed to acclimatize to the new environment inside the cage for 7 days prior to the start of the study, and the ears were punctured 3 days prior to confirmation.

#### MRI acquisition

MRI was performed using a 7.0 T small animal scanner (Biospin 70/20 USR; Bruker, Fällanden, Switzerland). A quadrature birdcage coil (inner diameter, 72 mm) was used for excitation, and an actively decoupled 4-channel phased array surface coil was used to receive the signal. T2-weighted images were acquired from C57BL/6N mice under isoflurane anesthesia (5% for induction, 1.5% for maintenance) using a turbo rapid acquisition with refocusing echoes (Turbo RARE) sequence with the following parameters: repetition time (TR)/echo time (TE) = 3000/60 ms; number of averages = 4; field of view = 30 × 30 mm^2^; image matrix = 192 × 192; and in-plane resolution = 0.156 × 0.156 × 0.75 mm^3^^19^.

#### Measuring intensity of magnetic field in air

Additionally, to confirm difference in output of rTMS coil for mouse form it of conventionally used for human application, the electric field intensities from those were compared by the same stimulation conditions at a place in air. As for human rTMS, a conventional clinical device, Brain-Stim-A of Remed Co. which acquired approval of Korean government was used.

### Statistical analysis

Data are presented as mean ± standard error. Statistical comparison between each group was performed on values calculated through simulation and magnetic field applied values by distance using one-way ANOVA using SPSS version 21.0 (IBM, Chicago, IL, USA). A value of p < 0.05 was considered statistically significant as different.

## Results

### Fabrication of new rTMS coil for murine head model

**Figure 12:**
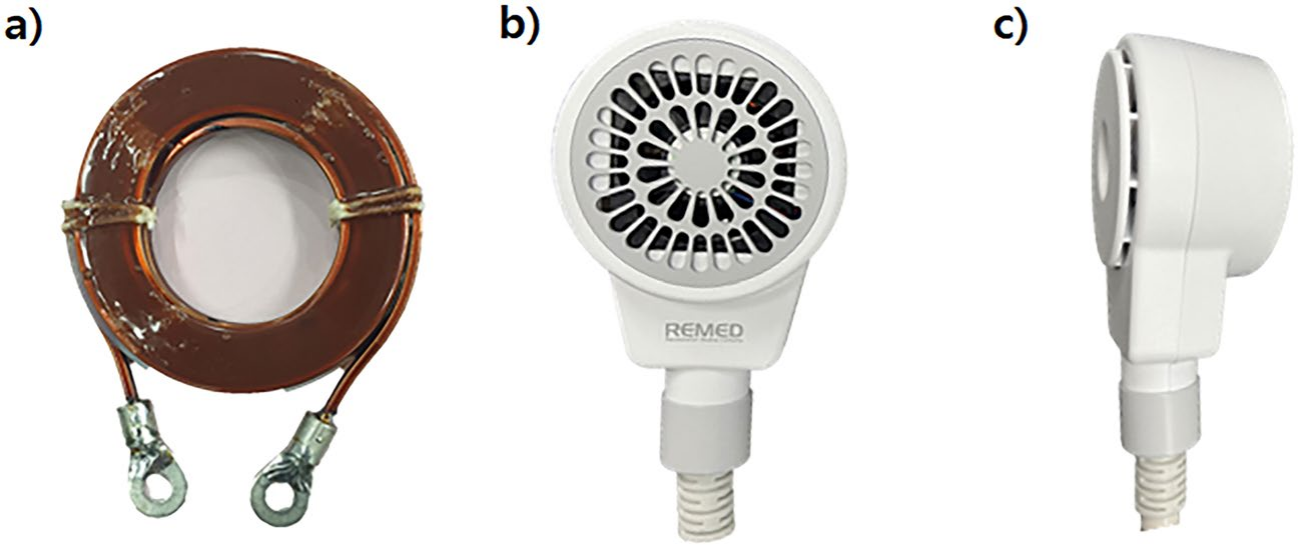
Fabrication of new rTMS coil for murine head model. (**a**) Manufactured coil according to the design; (**b**) front view; (**c**) side view of the manufactured prototype coil in air-cooled casing.

The coil was manufactured according to the simulation design shown in Fig. 2 (Fig. 12a). Due to close location of the coil on the head of each mouse, thermal stability was considered to ensure safety. Because of the small coil size, high current and high magnetic field results in thermal and mechanical instability, and the high currents used for rTMS could harvest hazardous levels of Joule heating that could injure an animal by overheating^13,14^. Accordingly, a unique coil case was manufactured for the designed TMS coil, a cooling fan was placed above the coil, and small vents were added on the top face of the case to dissipate heat (Fig. 12b,c) and the coil was kept cool during the therapy procedure.

**Figure 13:**
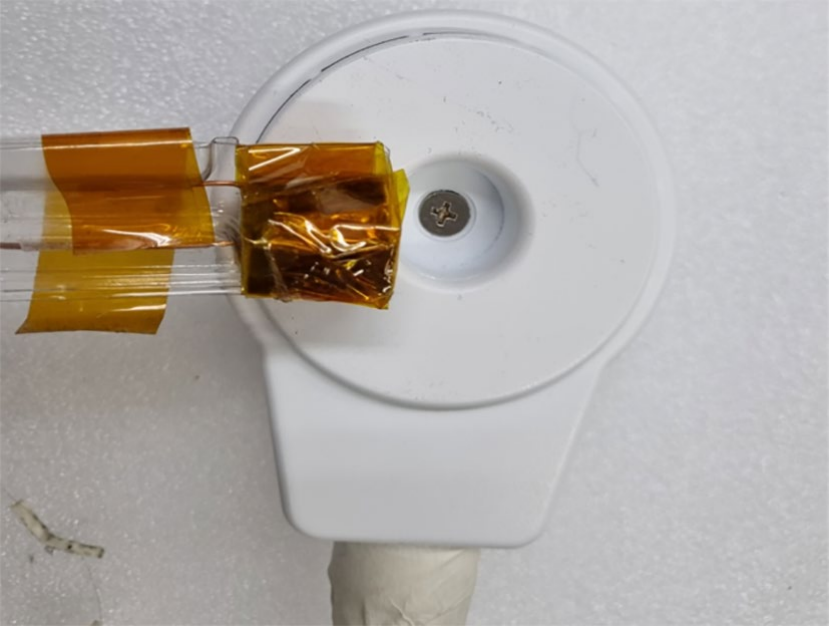
Measurement setup of electric field intensity of fabricated coil.

**Figure 14:**
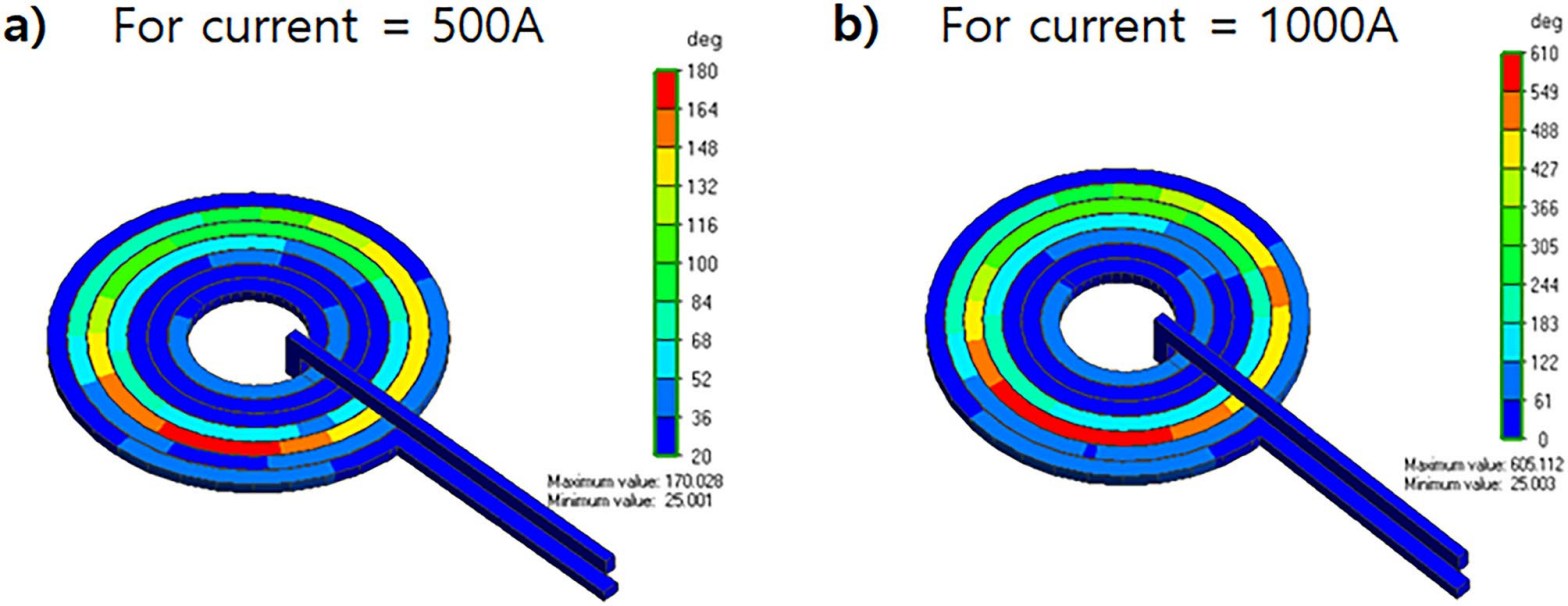
Thermal distribution of coil simulation using different current values: (**a**) 500 A; (**b**) 1000 A.

To verify the simulation data, values of magnetic field intensities from the simulation was compared from the measured values of in vivo experiments. Since rTMS is expected to stimulate cerebral cortex in clinical use, the measurement target was also cerebral cortex of alive mouse. Stimulation parameters of rTMS was the same as the simulated condition including current, voltage, frequency, and temperature of the atmosphere with 25 °C. The electric field intensity was measured with an E-field probe using PSD (power spectral density) method on the surface of the coil as shown in Fig. 13. Comparison analysis between simulated and measured values on the surface of the coil showed great agreement (Table 5). Furthermore, the coil was simulated to check the thermal profile with two different current excitations (500 A and 1000 A). The mean coil temperature is around 40 °C at an excitation of 500 A (Fig. 14a) and 65 °C at an excitation of 1000 A (Fig. 14b) was applied, which made the coil thermally stable.

**Table 5:** Electric field intensity summary.

Voltage	Simulated E-field intensity	Measured E-field intensity
500 V	136.1452 V/m	140.0 V/m

### Change in magnetic field according to coil type, distance, and in vivo measurement

**Figure 15:**
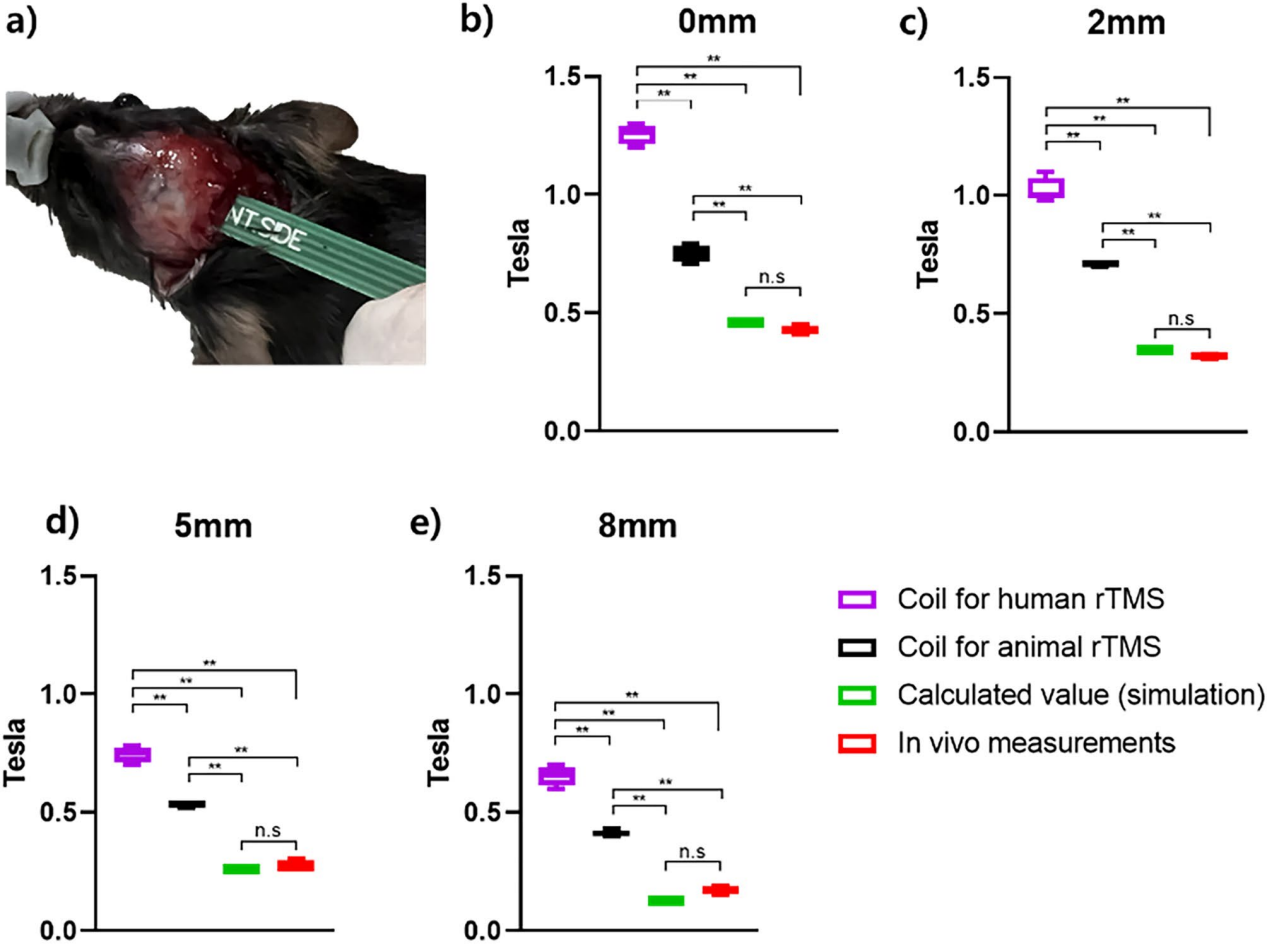
Prediction of magnetic field by repetitive transcranial magnetic stimulation (rTMS) type and measurement of magnetic fields in vivo*:* (**a**) in vivo experimental setup with the Tesla meter comparing the human coil, murine coil, simulation results, and experiment results with changes in distance between coil and head of: (**b**) 0 mm; (**c**) 2 mm; (**d**) 5 mm; and (**e**) 8 mm. Levels of significance for comparison by one-way ANOVA test: **p < 0.01, n.s, not significant. *p* = 0.448, 0 mm n.s; *p* = 0.518, 2 mm n.s; *p* = 0.8327, 5 mm n.s; *p* = 0.0509, 8 mm n.s.

To compare the conditions set in the simulation using the newly designed rTMS coil, a Tesla meter was inserted into the murine head and the distance between the coil and head adjusted and measured (Fig. 15a). The distances between the rTMS coil and the head were set to 0, 2, 5, and 8 mm and compared to those of the human coil used in the past. The human coil had the highest Tesla measured under all distance conditions, and a significant difference was noted from that of the murine coil. The magnetic field to be applied according to the distance was predicted through simulation, and there was no statistical difference between the predicted and measured values in vivo (Fig. 15b–e).

## Discussion

This study aimed to establish the existing figure 8-shaped rTMS coil and a miniaturized coil unique to rodents to establish their focus and heat stabilities. Additionally, the device was verified by predicting and measuring the in vivo magnetic field using an established coil based on computational simulations. Many studies have applied rTMS coils made for humans to animals, but the reliability of the data must be reconsidered^11^. Rodents are the most commonly used laboratory animals, but their brain structures differ in important ways from those of humans^20^. The smooth cortex has a very different geometry than the highly folded human cortex, which is an important consideration because the properties of the electric field induced by rTMS are assumed affected by the orientation of the tissue relative to the coil^21^. Moreover, the small size of the rodent brain is also a concern, as in most animal models, even the smallest commercially available rTMS coils have different head-to-coil size ratios than those in humans, resulting in a reduced stimulation concentration and efficiency^22^. The use of a figure 8-shaped coil in rodents can stimulate the entire brain and other parts of the body. Therefore, size discrepancies do not allow for easy interpretation and translation of animal results into clinical applications.

Many rodent studies used human-scale coils to deliver rTMS, while others used miniaturized rTMS coils to mimic focal human rTMS in rodents more closely^23–25^. Small coils are more advantageous for focusing murine versus human rTMS; however, because of thermal issues, their use should be limited to a level approximately 10–100 times lower than the strength of magnetic fields typically applied to humans^22,26^. Interestingly, rTMS was first reported 30 years ago in a study using a circular coil^1^ with which it was difficult to locally stimulate a target region of the brain. Next, a local brain stimulation method using a figure 8-shaped coil was proposed, and stimulation of the human motor cortex within 5 mm resolution was achieved^27^. Implementation of the rTMS using a figure 8-shaped coil is advantageous for local stimulation of the brain and widely used in basic and clinical medicine^28^. Despite the progress of various studies on rTMS and surface and deep brain stimulation according to the structural modification of the figure 8-shaped coil^29^, the two designs showed similar reproducibility based on recent clinical studies^30^. Thus, we created a miniaturized coil suitable for animals rather than a large coil; as a result of designing and simulating various shapes for focusing ability, we confirmed that the single coil showed stronger focusing ability than the figure 8-shaped coil. In addition, by investigating the stability, such as that of the coil's heat generation, an optimal design was created, to which an air cooler was added to develop a prototype capable of providing efficient thermal control.

To construct an accurate animal head model, a numerical simulation model of the animal was created by digitizing the animal head structure using 7.0 T MRI^19^. The coil developed by this research team was applied to an animal head model to measure the actual magnetic field value, and the conditions for applying the voltage under the same conditions as those of the clinical study were traced. The experimental results confirmed that when a magnetic field was applied to the human head simulation model using the rTMS coil design under various conditions, it was more reliably focused on the coil of the circular design. This is probably due to the use of a simulation technique that differed from other studies; however, the optimal coil design study should be conducted under various conditions.

Finally, comparison of the applied magnetic field values of the large and miniaturized coil revealed large magnetic field differences under all conditions. Moreover, the measurements using the Tesla meter on the head of the actual animal were surprisingly nearly identical to those predicted by the simulation model. This study overcame the limitations of rTMS studies and modeled them in more detail. Nevertheless, it had several limitations. First, many strains of murine phantoms could not be obtained, and only the C57BL6 murine head model was simulated. Second, no other programming tools were used for the TMS coil simulation. Third, a Tesla meter was used to measure the magnetic field, but a more accurate platform was required. However, our study is expected to provide more reliable data based on animal experiments that are identical to clinical conditions. Our research using this biomimetic platform will be actively utilized in future intra-brain research of rTMS.

## Data Availability

The data presented in this study are available upon request from the corresponding author.
